# Pharmacogenetic landscape of Metabolic Syndrome components drug response in Tunisia and comparison with worldwide populations

**DOI:** 10.1371/journal.pone.0194842

**Published:** 2018-04-13

**Authors:** Haifa Jmel, Lilia Romdhane, Yosra Ben Halima, Meriem Hechmi, Chokri Naouali, Hamza Dallali, Yosr Hamdi, Jingxuan Shan, Abdelmajid Abid, Henda Jamoussi, Sameh Trabelsi, Lotfi Chouchane, Donata Luiselli, Sonia Abdelhak, Rym Kefi

**Affiliations:** 1 Laboratory of Biomedical Genomics and Oncogenetics, Institut Pasteur de Tunis, Tunis, Tunisia; 2 University of Carthage, Tunis, Tunisia; 3 University of Tunis El Manar, Tunis, Tunisia; 4 Laboratory of Genetic Medicine and Immunology, Weill Cornell Medical College in Qatar, Qatar Foundation, Doha, Qatar; 5 Department of external consultation, National Institute of Nutrition and Food Technology, Tunis, Tunisia; 6 Clinical Pharmacology Service, National Pharmacovigilance Center, Tunis, Tunisia; 7 Laboratory of Molecular Anthropology, Department of Biological, Geological and Environmental Sciences (BiGeA), University of Bologna, Bologna, Italy; Universita degli Studi di Roma Tor Vergata, ITALY

## Abstract

Genetic variation is an important determinant affecting either drug response or susceptibility to adverse drug reactions. Several studies have highlighted the importance of ethnicity in influencing drug response variability that should be considered during drug development. Our objective is to characterize the genetic variability of some pharmacogenes involved in the response to drugs used for the treatment of Metabolic Syndrome (MetS) in Tunisia and to compare our results to the worldwide populations. A set of 135 Tunisians was genotyped using the Affymetrix Chip 6.0 genotyping array. Variants located in 24 Very Important Pharmacogenes (VIP) involved in MetS drug response were extracted from the genotyping data. Analysis of variant distribution in Tunisian population compared to 20 worldwide populations publicly available was performed using R software packages. Common variants between Tunisians and the 20 investigated populations were extracted from genotyping data. Multidimensional screening showed that Tunisian population is clustered with North African and European populations. The greatest divergence was observed with the African and Asian population. In addition, we performed Inter-ethnic comparison based on the genotype frequencies of five VIP biomarkers. The genotype frequencies of the biomarkers rs3846662, rs1045642, rs7294 and rs12255372 located respectively in *HMGCR*, *ABCB1*, *VKORC1 and TCF7L2* are similar between Tunisian, Tuscan (TSI) and European (CEU). The genotype frequency of the variant rs776746 located in *CYP3A5* gene is similar between Tunisian and African populations and different from CEU and TSI. The present study shows that the genetic make up of the Tunisian population is relatively complex in regard to pharmacogenes and reflects previous historical events. It is important to consider this ethnic difference in drug prescription in order to optimize drug response to avoid serious adverse drug reactions. Taking into account similarities with other neighboring populations, our study has an impact not only on the Tunisian population but also on North African population which are underrepresented in pharmacogenomic studies.

## Introduction

Drug response varies between individuals owing to disease heterogeneity, genetic and environmental factors [1–3]. It depends on absorption and distribution of drug to the targeted receptors and enzymes that further metabolize it and ultimately excrete it from the body [4]. During this process, genetic variation may alter the therapeutic response of an individual [5–7]. Indeed, ethnic diversity plays a major role in drug response variability, which may have an important regulatory aspect that should be also considered during drug development [8]. Consequently, treatment and recommended doses should not be extrapolated from one ethnic group to another [9]. Pharmacogenetic studies explore the genetic impact of the inter-individual variability of drug response, involving both the pharmacokinetics and pharmacodynamics [3]. The clinical implementation of pharmacogenetics in therapeutic approaches, aims to optimize specific drug regimens and drug dosage. This, may have a great advantage in, improving clinical outcomes and avoiding major clinical complication such as congestive heart failure, hepatic and renal disorders [10, 11].

During the last years, the incorporation of pharmacogenetic testing in clinical trials has gained interest. This has been facilitated with the advancement in microarray based methods that allows genotyping Single Nucleotide Polymorphisms (SNPs) in many samples simultaneously. This method leads to the identification of loci responsible for drug response variability and adverse reactions [12, 13]. Despite the double burden of communicable and non communicable diseases (NCDs) on the health system, some populations like African still remain poorly studied at the pharmacogenetic level. The Metabolic Syndrome (MetS) is one of the diseases which prevalence is increasing dramatically in North Africa. In Tunisia, it affects up to 30% of the population in urban region [14]. MetS is defined by a cluster of multiple metabolic abnormalities, including central obesity, hypertension, dyslipidemia and insulin resistance that directly increase the risk of coronary heart disease (CHD), other forms of cardiovascular atherosclerotic diseases (CAD), Type 2 Diabetes Mellitus (T2DM), and premature death [13, 15]. Until now, there is no effective drug treatment prescribed to manage all components of MetS. Indeed each syndrome component is treated individually, so miscellaneous types of drugs are used in the treatment of this syndrome, including weight losing drugs, antilipemic, antihypertensives and antidiabetics [15]. This strategy is not always reliable; indeed the individuals do not respond equally to the same treatment. Such differences may be due to diverse mean values of quantitative traits or different genotypic frequency distributions of the variants between populations [16]. For instance, many studies have reported the involvement of variants annotated as Very Important Pharmacogene (VIP) variants in the outcome of anticoagulant, oral anti-diabetic [17, 18] and statins [19]. A significant proportion of ethnic variability in the response to coumarin anticoagulant (AVK), prescribed to treat and prevent arterial and venous thromboembolic disorders in MetS, has been attributed to the differential frequencies of variant in *CYP2C9* and *VKORC1* genes[20, 21]. The diversity of distribution of functional variants, rs9923231 located in *VKORC1*, rs1799853 and rs1057910 located in *CYP2C9*, and which are known to influence AVK dose requirement, has been highlighted [21]. These common polymorphisms were shown to confer a high risk towards over-anticoagulation, predisposing the individuals carrying these polymorphisms to hemorrhagic incidents [22]. In addition, a variable response to beta-blockers used in the treatment of hypertension, including propranolol [23], metoprolol, atenolol [24] and clopidogrel [25, 26] was observed in patients of European and African origins which can trigger a stroke [27, 28]. Other serious adverse outcomes of drugs in MetS are reported such as sudden death, bleeding events, myopathy, [2, 20, 27, 29]. All these ADR engender high social and financial burden. Considering the burden of public health, and genetic variability in response to treatment, we have chosen in the present study to characterize the genetic variability of some pharmacogenes involved in the response to drugs used for the treatment of MetS in Tunisia and to compare our results to the worldwide human populations.

## Materiel & methods

### Study participants and genotyping

A set of 135 Tunisian healthy individuals including; 32 women and 103 men, originating from two regions (the Capital Tunis and the coastal city of Monastir) were genotyped in a previous study [30] using the AffymetrixChip 6.0 genotyping array. Genotyping data were generated after the variants calling performed with R CRLMM package [31]. The study was approved by the Ethics Committee of the Institut Pasteur (Tunis, Tunisia-Registration numbers IRB00005445, FWA00010074, and PV09/06, IRB# 0000000044). All participants provided written informed consent.

### Selection of very important pharmacogenes and variant

We selected a set of very important pharmacogenes (VIP) involved in MetS components drug response from the PharmGKB database (http://www.pharmgkb.org) which provides an overview of significant genes involved in the metabolism or response to one or several drugs. VIP genes were chosen through extensive review of a variety of sources, including the U.S. Food and Drug Administration (FDA) biomarker list, FDA-approved drug labels with pharmacogenetic information, and Clinical Pharmacogenetic Implementation Consortium (CPIC) nominations [32]. Additionally, we considered a gene as a VIP if it is associated with a large number of variant annotations and having high-level of clinical annotations. Furthermore, genetic variants were chosen from published polymorphisms associated with VIP through an extensive bibliographic search.

### Genotyping data

PLINK v2 [33] was used to extract variants of the selected VIP. The genotypic data of individuals from 22 other populations were downloaded from the International HapMap Project phase III (ftp://ftp.ncbi.nlm.nih.gov/hapmap/) and published data [34, 35]. The studied populations included those of (1) African ancestry in the South Western USA (ASW); (2) Northwestern and western European ancestry populations of Utah from the CEPH collection (CEU); (3) Han Chinese in Beijing, China (CHB); (4) Chinese population of metropolitan Denver, Colorado, USA (CHD); (5) Gujarati Indians in Houston, Texas, USA (GIH); (6)Luhya people in Webuye, Kenya (LWK); (7) people of Mexican ancestry living in Los Angeles, California, USA (MEX); (8) Maasai people in Kinyawa, Kenya (MKK); (9) Toscani people of Italy (TSI); and (10)Yoruba in Ibadan, Nigeria (YRI).

Genotypic data from other population mainly from South Europe and North Africa (Algeria (ALG), Egypt (EGY), Libya (LIB), Tunisia Douiret (TN_Ber), Lebanon (LEB), South Morocco (MCS), North Morocco (MCN), Sub-Saharan (SAH), South Spain (SPS), North Spain (SPN) Spain Basc (SBA) were download from the previous published study [16, 35].

### Quality control analysis

Genotypic data were managed using the PLINK v2 software. Variants were excluded if they are deviating from the Hardy-Weinberg equilibrium (HWE) (p-value < 10^−4^), minor allele frequency (MAF) < 10^−2^ and have missed genotyping rate ≥ to 95% for each of the studied populations.

### Statistical analysis

To infer cryptic population structure from genomic data, principal component analysis (PCA) based on pruned genotypic data was performed using SNPrelate R package [36]. Cryptic population structure defines a population structure that is difficult to detect using visible characters but may be significant in genetic terms[37, 38]. Indeed, the information about the population origin is given by the study participants. This information is subjectively based on geographic location, physical and cultural characters. The genotypic data and estimated allele frequencies might be used to determine if a given assignment of individuals to a population, based on subjective criteria, mirrors a natural assignment in genetic terms (admixture and gene flow). Genetic data can be useful to determine the cryptic relatedness among populations and to shape the false matches due to the probabilistical assignment of population. In addition, we used PLINK v2, and “rgl” R package [39], to generate multidimensional scaling plot (MDS) and three dimensional MDS (3D MDS) from the same data.

The genotype frequencies of the selected VIP in Tunisian population, were calculated and compared with 10 HapMap populations, using SNPassoc R package [40]. In this step of analysis, we have considered only the HapMap project populations due to the complete genotypic data. The inter-ethnic genotypic frequency comparison was performed using the Chi-square test and Bonferroni’s adjustement was applied to the level of significance set at a p-value threshold of 5% devided by the number of studied loci.

### Analyses of population genetic structures

Fixation Index (Fst) and Structure are two common analyses in population genetic studies. The R package Hierfstat [41] was used in order to assess the degree of similarity in genetic structure between the different ethnic populations, we calculated pairwise Fst values and evaluate the magnitude of differentiation among geographic populations (0 indicating no divergence, 1 meaning complete separation). Pairwise Fst values between the Tunisian population and the other 10 HapMap populations were calculated. To further investigate variation at the VIP variants in terms of population structure, we used the STRUCTURE ver. 2.3.4 software [38, 42] which is based on the bayesian clustering algorithm to assign the samples within a hypothetical K number of ancestries. We set a range of possible number of clusters ranging from K = 2 to K = 10 and 24 trials were run for each K. The Markov Chain Monte Carlo iteration for each structure analysis was run for 10000 after an initial burn-in period of 10000 steps. In order to assess the most likely number of clusters, we calculated delta K as proposed by Evanno et al. [43]. The similarity of the runs at each K level was evaluated by the CLUMPP software as implemented at the online [44]. The Distruct software was used to visualize the best alignment of subpopulation inferring population substructure and individual assignment across the best runs at each k level [45].

## Results

Based on a large bibliographic search and PhamaGKB interrogation, we selected 24 pharmacogenes implicated in MetS components drug response modulation listed in Table 1 including class of drug, drug name, gene name, description and category family of genes, pharmacokinetic phase of drug metabolism, chromosomal localization and the corresponding VIP variant. The studied genes belong to ABC transporters family, cytochrome P450 family and G-coupled receptor family (Table 1). A total of 1056 variant on 24 pharmacogenes were identified in the Tunisian population and kept after quality control steps for subsequent analysis.

**Table 1:** Basic information of selected pharmacogenes.

Class of Drug	Drug name	Genes ID	Description	Category Family	Phase	Chr	Localisation of Gene	VIP (High evidence level)
Anticagulant	Clopidogrel (Palvix)	*ABCB1*	ATP binding cassette subfamily B member 1	ABC transporters superfamily	Other	7	chr7:87,133,179–87,342,638	rs10545642 rs1128503 rs2032582
		*CYP2D6*	cytochrome P450 family 2 subfamily D member 6	Cytochrome P450 superfamily	phaseI	22	chr22:42126499–42130881	rs3892097
		*CYP2C19*	cytochrome P450 family 2 subfamily C member 19	Cytochrome P450 superfamily	phaseI	10	chr10:96,522,438–96,612,962	rs1057910 rs4244285
		*P2RY12*	purinergic receptor P2Y, G-protein coupled, 12	G-protein coupled receptor	Other	3	chr3:151,054,631–151,102,600	
	Acenocoumarol	*CYP2C9*	cytochrome P450 family 2 subfamily C member 2	Cytochrome P450 superfamily	phaseI	10	chr10:96,698,415–96,749,148	rs1057910
		*VKORC1*	vitamin K epoxide reductase complex, subunit 1	Vitamin K epoxide reductase	phaseI	16	chr16:31,102,163–31,106,320	rs9934438 rs7294
Antidiabetic	Biguanide/Metformine	*SLC22A1*	solute carrier family 22 (organic cation transporter), member 1	Organic cation transporte	other	6	chr6:160,542,863–160,579,750	
	Biguanide/Metformine	*SLC22A2*	solute carrier family 22 (organic cation transporter), member 2	Organic cation transporte	other	6	chr6:160,637,794–160,679,963	
	Biguanide/Metformine	*SLC47A1*	solute carrier family 47 (multidrug and toxin extrusion), member 1	Multidrug and toxin extrusion	other	17	chr17:19,437,167–19,482,346	
	Biguanide/Metformine	*SLC47A2*	solute carrier family 47 (multidrug and toxin extrusion), member 2	Multidrug and toxin extrusion	other		chr17:19,581,628–19,620,043	
	Biguanide/Metformine	*ATM*	ATM serine/threonine kinase	Phosphatidylinositol 3-kinase-related kinase superfamily	Other	11	chr11:108,093,559–108,239,826	rs11212617
	TZD/Pioglitazone	*PPARG*	peroxisome proliferator-activated receptor gamma	Nuclear receptors superfamily	Other	3		
	TZD/Rosiglitazone	*PGC-1alpha*	peroxisome proliferative activated receptor, gamma, coactivator 1 alpha	Nuclear receptors superfamily	Other	5	chr5:51,454,249–51,553,921	rs1801282
	TZD/Troglitazone	*RETN*	Resitin			19	chr19:7,669,086–7,670,454	
		*Leptin LEPR*	leptin receptor	Cytokine receptors superfamily	Other	1	chr1:65,420,652–65,635,428	
		*TNFalpha*	Tumor Necrosis Factor	Tumor necrosis factor receptor		31	chr6:31,543,344–31,546,112	
	Sulphonylurea	*KCNJ11*	potassium channel, inwardly rectifying subfamily J, member 11	Potassium channel	Modifier	11	chr11:17,406,796–17,410,206	rs5215 rs5219 rs757110
		*ABCC8*	ATP binding cassette subfamily C member 8	ATP-binding cassette (ABC) transporters	Modifier	11	chr11:17,414,432–17,498,392	
		*KCNQ1*	potassium channel, voltage gated KQT-like subfamily Q, member 1	Potassium Channel superfamily	Other	11	chr11:2,466,221–2,870,340	
		*TCF7L2*	transcription factor 7-like 2 (T-cell specific, HMG-box)	DNA-binding proteins.	Other	10	chr10:114,710,009–114,927,436	rs12255372
Lipid lowring Fenofibrate	Flavastatin	*CYP2C9*	Cytochrome P450 family 2 subfamily C member 9	Cytochrome P450	phaseI	10	chr10:96,698,415–96,749,148	
	Lovastatin	*ABCB1*	ATP binding cassette subfamily B member 1	ABC transporters superfamily	Other	7	chr7:87,133,179–87,342,638	rs1128503
	Atorvastatin	*ABCB1*	ATP binding cassette subfamily B member 1	ABC transporters superfamily	Other	7	chr7:87,133,179–87,342,638	rs2032582,
		*ABCA1*	ATP-binding cassette, sub-family A (ABC1), member 1	ABC transporters sub-family A	Other	9	chr9:104,781,003–104,928,246	rs12003906
		*CYP2C9*	Cytochrome P450 family 2 subfamily C member 9	Cytochrome P450	phaseI	10	chr10:96,698,415–96,749,148	rs1057910
		*PPARA*	Peroxisome proliferator-activated receptor alpha	Nuclear hormone receptor superfamily		22	chr22:46,546,499–46,639,653	
		*SCLO1B1*	Solute carrier organic anion transporter family, member 1B1	Solute carrier family	Others		chr22:46,546,499–46,639,653	rs4149056 rs4149081 rs4363657 rs4149015
		*HMGCR*	3-hydroxy-3-methylglutaryl-CoA reductase	HMGCR superfamily		5	chr5:74,632,993–74,657,926	rs17238540 rs3846662 rs17244841
		*CYP3A5*	cytochrome P450 family 3 subfamily A member 5	Cytochrome P450 superfamily	Phase I	7	chr7:99,245,812–99,277,649	rs776746

The list of 24 pharmacogenes implicated in MetS components drug response modulation, including class of drug, drug name, gene name, description and category family of genes, pharmacokinetic phase of drug metabolism, chromosomal localization and the corresponding common VIP variant.

**Fig 1:**
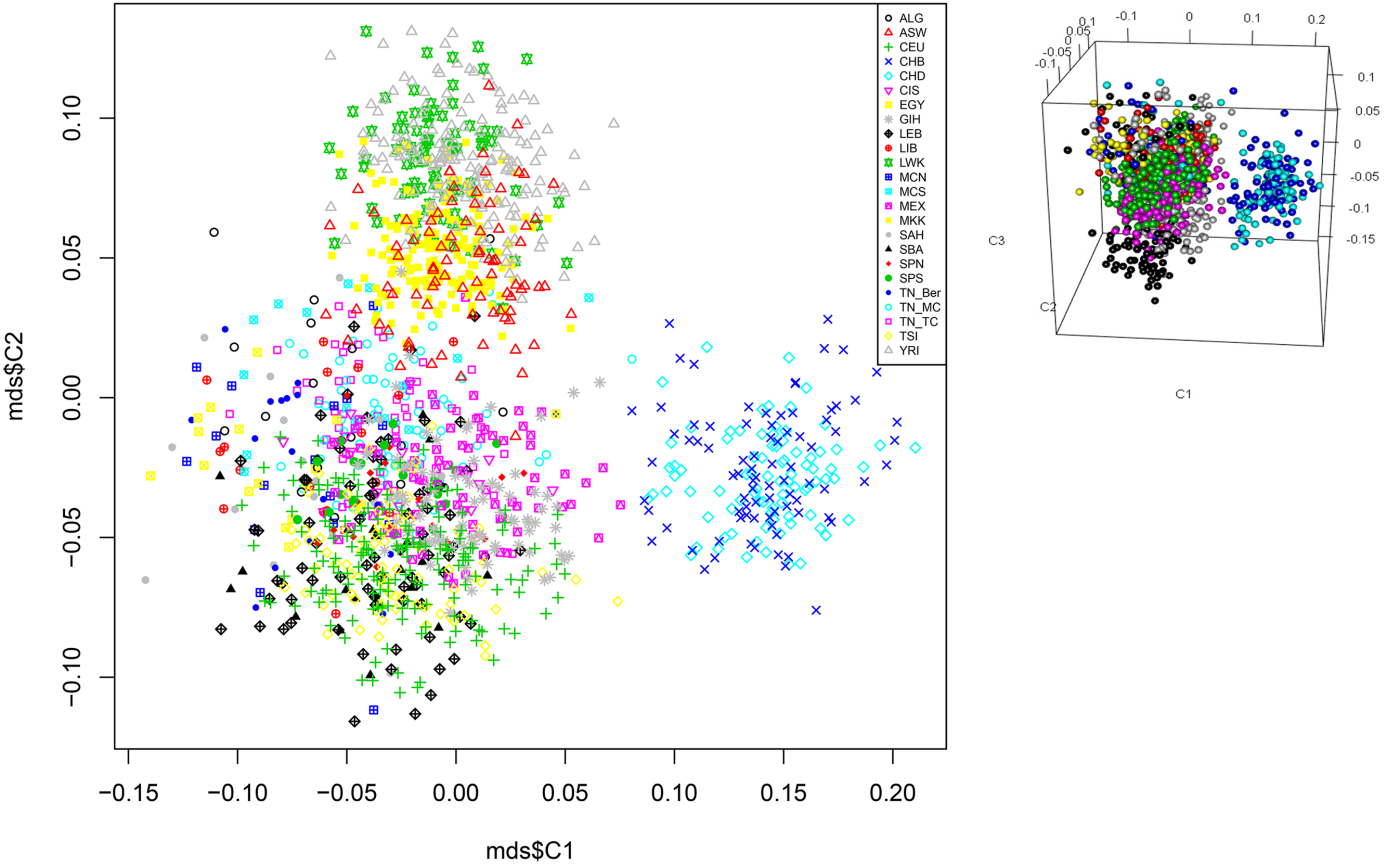
Multidimensional scaling plot analysis of the Tunisian subpopulation and worldwide populations. The plot reveals three distinct clusters showing that the Tunisian population present a close affinity with the North Africans and Europeans and distinct from South Africans and Asians. Tunisian population; Capital Tunis TU_TC, coastal city of Monastir TU_MC (AffymetrixChip 6.0 genotyping array), African ancestry in the south Western USA (ASW); a northwestern European population (CEU); the Han Chinese in Beijing, China (CHB); a Chinese population of metropolitan Denver, Colorado, USA (CHD); the Gujarati Indians in Houston, Texas, USA (GIH); the Japanese population in Tokyo, Japan (JPT); the Luhya people in Webuye, Kenya (LWK); people of Mexican ancestry living in Los Angeles, California, USA (MEX); the Maasai people in Kinyawa, Kenya (MKK); the Tuscan people of Italy (TSI); and the Yoruba in Ibadan, Nigeria (YRI); data from HapMap were retrived in March 2016. It is available by FTP: ftp://ftp.ncbi.nlm.nih.gov/hapmap/ and Algeria (ALG), Egyptia (EGY), Libya (LIB), Tunisia Dwiret TUN_Ber, Lebanon (LIB), Morocco South (MCS), Morocco North (MCN), Spain South (SPS), Spain North (SPN), Spain Basc (SBA),: Sub-Saharan (SAH), Canary Island (CIS); data from the literature [34, 35].

For inter-ethnic genotypic frequency comparison and population structure analyses purposes, we kept 743 shared variants among the 22 worldwide studied populations. The MDS analysis describing the genetic landscape of these pharmacogenes shows a cluster of the Tunisian population with the North African populations (Algeria, Morocco, Egypt …), Tuscan and CEU were distinguished from the Asian and Sub Saharan African populations (Fig 1). This result was further confirmed using PCA analyses (S1 Fig). A great divergence was observed between the Asian populations and LWK (S1 Fig).

Among the 743 variants shared between the studied populations, five clinically relevant VIP variants were identified. Therefore, we performed an inter-ethnic comparison based on their genotypic frequencies. We found that the genotype frequencies of the following variants involved in the anticoagulant sensitivity; rs3846662 (*HMGGR*), rs1045642 (*ABCB1*), rs7294 (*VKORC1*) and rs12255372 (*TCF7L2*), were similar between the Tunisian population, Tuscany and the European populations (CEU); (p>>0.05/5*10). The genotypic frequencies of the rs776746 variant located in the *CYP3A5* gene involved in the hypolipidemic susceptibility are similar between Tunisian and African populations (MKK, LWK, YRI) and significantly different from European (CEU) and Asian (CHD, CHB); (p<<0.05/5*10) (Table 2).

**Table 2:** Genotype frequency of significant VIP variants in Tunisian population (n = 135) compared with ten HapMap populations.

Gene name	SNP ID	Freq.(TUN)*			p-values against ten populations after Bonferroni correction									
		AA	AB	BB	CEU	TSI	ASW	LWK	MKK	YRI	CHB	CHD	GIH	MEX
***CYP3A5***	rs776746	0;66	0;26	0;08	1.403e^-05^	0.0001	0.0056	**0.1242**	**0.0952**	**0.1507**	**0.04827**	**0.1836**	**0.2151**	**0.2151**
***HMGCR***	rs3846662	0.29	0.47	0.25	**0.4508**	**0.8194**	7.205e^-12^	2.2e^-16^	8.61e-^10^	2.2e^-16^	**0.9271**	**0.8383**	**0.0226**	**0.2502**
***ABCB1***	rs1045642	0.49	0.33	0.18	**0.0262**	**0.0152**	0.0007	-	0.0004	6.802e^-06^	**0.4246**	0.0066	0.0066	0.0002
***VKORC1***	rs7294	0.65	0.27	0.09	**0.0902**	**0.0014**	**0.0013**	4.081e^-07^	3.722e^-06^	2.226e^-06^	6.742e^-05^	0.0005	0.0001	0.0013
***TCF7L2***	rs12255372	0.48	0.33	0.19	**0.0487**	**0.0264**	**0.0071**	**0.0071**	**0.0236**	**-**	**0.02361**	4.248e-^10^	0.0002	**0.0216**

Bonferroni correction was applied to the level of significance, which was set at (p.value < 0.05/5*10 = 0.001);p.value > (0.05/5*10) assigned in bold represent differences not statistically significant between Tunisia and the compared population, 0.00 not observed genotypes; -: not successfully genotyped or not compared by chi-square test. TUN* = (TN_Mc/TN_TC), MEX = Mexican ancestry in Los Angeles, California; TSI = Toscans in Italy; LWK = Luhya in Webuye, Kenya; ASW = African ancestry in Southwest USA; GIH = Gujarati Indians in Houston, Texas; MKK = Maasai.

In order to assess the degree of similarity in the genetic structure between the different ethnic populations, we calculated the pairwise Fst values among the Tunisian population and the other 10 HapMap populations ranged from 0.00802 to 0.41201 (Table 3). Comparing Tunisia to other populations, the lowest level of differentiation was observed between the inner and costal part of centeral Tunisia (Fst = 0.01182), followed by the TSI (Fst = 0.02872) and MEX (Fst = 0,0269) populations, whereas the greatest divergence was observed with the LWK population (Fst = 0,35929).

**Table 3:** Estimating of pairwise Fst among the 11 populations.

	TN_TC	TN_MC	ASW	CEU	CHB	CHD	GIH	LWK	MEX	MKK	TSI	YRI
TN_TC	0											
TN_MC	0.01182	0										
ASW	0.15553	0.23478	0									
CEU	0.06517	0.04978	0.29914	0								
CHB	0.02268	0.02350	0.21765	0.09797	0							
CHD	0.06546	0.09602	0.26864	0.11989	0.00179	0						
GIH	0.10636	0.14372	0.17866	0.08929	0.18313	0.21073	0					
LWK	0.24521	0.32005	0.02744	0.35559	0.27347	0.32958	0.22448	0				
MEX	0.02691	0.02635	0.2041	0.031436	0.04645	0.06478	0.08524	0.27337	0			
MKK	0.12447	0.21536	0.03173	0.25203	0.18501	0.18924	0.15348	0.09203	0.16398	0		
TSI	0.02872	0.01614	0.26226	0.00802	0.06736	0.11424	0.09639	0.34295	0.02816	0.22575	0	
YRI	0.27506	0.35929	0.01749	0.41201	0.32575	0.37106	0.29278	0.00276	0.32288	0.08404	0.38693	0

The pairwise differences Fst values between 11 populations. Fst value is less than 0.15 represent that there is no genetic differentiation between the two populations. The lowest level of differentiations were found between TN_TC and TN_MC (Fst = 0,01182) followed by the TSI (Fst = 0.02872) and MEX (Fst = 0,0269) populations, whereas the greatest divergence was observed with the LWK population (Fst = 0,35929).

**Fig 2:**
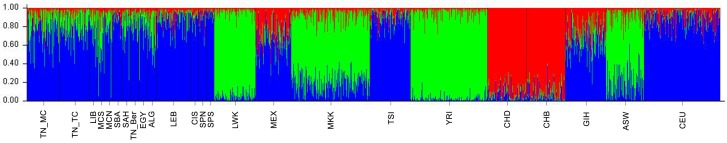
STRUCTURE analysis of the genetic relationship between 24 populations. K is the possible numbers of parental population clusters. One color represents one parental population into different color segments. Best K level was observed at K = 3, where a vertical the proportion of each ancestral component in a single individual is represented by a vertical bar divided into 3 colors. 601 markers study—displaying results for runs with highest likelihood out of 27 runs in each cluster K3 to 10. Black vertical lines identify the population boundaries. The height extent of each color within an individual’s color bar corresponds to the estimated membership of the individual in one of the clusters; each cluster is assigned a separate **color**. The bars with multiple colors can be interpreted as genetic admixture or as relative probabilities of belonging to the different clusters.

Bayesian-based STRUCTURE analysis (Fig 2) provided complementary methods for visualizing patterns of genetic similarity and differentiation between the Tunisian population and the other 22 populations. According to the Evanno’s ΔK method for STRUCTURE, K = 3 was selected to detect the most likely number of genetic clusters (S2 and S3 Figs). The barplot shows three components: African, Asiatic and European. For the North African populations cluster regrouping (TN_MC, TN_TC, ALG, EGY, MCN, MCS, SAH and LIB), the European; (CEU, TSI, MEX, SPB, SPN, SPS), East African (MKK, LWK, ASW) and Asiatic subgroups (CHD, CHB). This graph demonstrates the predominance of the African and European components. Thus, reflecting that the Tunisian population is a mosaic of different populations which reflects the existence of different gene flows that have influenced genetic variability of the response to treatments.

## Discussion

In the recent years, the use of pharmacogenomics witnessed important success in the improvement of healthcare by developing therapeutic treatment and predicting individual response [23]. Distribution of VIP variants exerts irreplaceable significance in pharmacogenomics knowledge [25]. In the present study, we showed that some VIP variants involved in MetS drug response, exhibited a great genetic variation among the studied populations, which directly impacts on the delivery of individualized medicine. The ethnicity should be taken into consideration, in routine clinical practice to ensure the efficacy and safety of the drug at the population level [46][47]. Our data confirm that ethnicity, even among close populations plays a significant role in differential distribution of variants implicated in drug response or ADR [46]. In Tunisian population, these variants displayed a close genetic affinity with the North African (ALG, LIB, MCN, MCS), Middle Eastern (EGY, LEB) and European populations (TSI, CEU, SPS, SBA, SPN) but they were distinct from the South Africans and Asians. Our results are in agreement with those of the study of Mizzi C et al, 2016, that used tailored DMET array and compared the generated data of 11 European populations against Saudi Arabian, Asian and South African populations. The study showed that there are no significant differences among the European populations. The great divergence was observed among European, Asian and South African population [46]. Another study of Abdelhedi et al., focused on the *CYP2C9* and *CYP2C19* variants implicated in the metabolism of anticoagulant response, concluded that Tunisians were similar to Europeans and Middle Easterners with regard to the allelic frequencies [27]. Pairwise Fst values of clinically relevant VIP variants, in our study, also revealed a more similarity between Tunisian, Tuscan and European populations (CEU). This was confirmed with analysis using admixture that showed the heterogeneity of Tunisian population and the contribution of the European, North African components. These observations were also reported on mitochondrial DNA, Y chromosomal and autosomal markers and interpreted as influences from different migration events [34, 48–50]. Obviously, differences in admixture history exert an important impact in the allelic and genotypic distribution of variants at the population level [47]. In the present study, five polymorphisms characterized as clinically relevant VIP variant, were selected, based on previous pharmacogenomics research to be further investigated (S4 Fig).

The variant rs7294; 3730 G>A transition located on chromosome 16 in the 3’Untranslated Region (UTR) of *VKORC1* gene. This gene encodes the vitamin K epoxide reductase protein, which is a crucial enzyme in vitamin K cycle and therefore involved in inter-individual drug variability of the majority of coumarin derivatives, such as warfarin, acenocoumarol and phenprocoumon which are frequently prescribed as oral anticoagulants to treat and prevent thromboembolism [51]. Because there is a large inter-individual and intra-individual variability in dose-response and a narrow therapeutic window, treatment with coumarin derivatives is challenging. Some polymorphisms in *VKORC*1 were associated with lower dose requirements and a higher risk of bleeding [52]. Patient with the TT genotype may require an increased dose to attend the curative effect of the anti-coagulant such as phenprocoumon or acenocoumarol as compared to patients with the TC or CC genotypes[53]. The frequency of TT genotype of rs7294 was generally lower in Tunisian population (0.09) than in African populations (MKK, LWK, ASW, YRI) respectively equal to (0.18, 0.28, 0.23) and East Asian populations (CHB, CHD) (0.89, 0.86) and similar to Europeans (CEU, MEX, TSI) (0.13, 0.09, 0.08) and Central Asians (GIH) (0.03) (S1 Table). Indeed, this result suggests that the Tunisians might require a lower dose of acenocoumarol or phenprocoumcon to achieve the therapeutic effect. Thus, the dosage regimen may be optimized on the specific genotypic frequency in Tunisian population.

The rs1045642 (A>G) transition is synonymous variant located on *ABCB1* (*MDR1*) gene which is one of many ubiquitous adenosine triphosphate (ATP)-binding cassette (ABC) genes that is responsible for cellular homeostasis [54, 55]. The *ABCB1* C3435T (rs1045642) is extensively studied and some research showed that the *ABCB1* C3435T genotype influences the absorption of clopidogrel [56] and is associated with poor clopidogrel response. Conversely, the frequencies of AA and AG genotypes of rs1045642 which respectively equal to (0.49, 0.33), in Tunisian population, were respectively much lower than that are reported in other populations. (S1 Table). In this case, AA and AG genotypes may have an increased risk of major adverse cardiovascular events such as cardiovascular death, myocardial infarction, or stroke, when treated with clopidogrel in people with acute coronary syndrome or myocardial infarction as compared to people with GG genotypes.

The rs3846662 located on intron 13 of *HMGCR*, was associated with differential induction, upon simvastatin exposure, of expression of full-length HMGCR transcript versus alternatively spliced transcript lacking exon 13 (HMGCRv_1). Homozygous individuals A/A exhibit 40% greater induction of full-length transcripts and 20% less alternatively spliced HMGCRv_1 transcript relative to A/G or G/G subjects [57]. These differences may have implications for simvastatin efficacy, since the AA genotype of rs3846662 was associated with the increased induction of the alternatively spliced transcript is correlated with reduced response to simvastatin [57]. For this variant, the Tunisian and European populations were genetically different to the African population (MKK, LWK, YRI) which represented a highly frequencies of the defective AA genotype which consisted respectively to the values of (0.93, 0.71, 0.92). These results mirror that the African populations were more sensitive to the statin treatment than Tunisian population which seems having a response similar to the European (TSI, CEU) (0.35, 0.32) (S1 Table).

Rs776746 (C/T) variant located on *CYP3A5* gene which is implicated in the biotransformation of the statin drugs, this variant creates a splice site in intron 3, resulting in altered mRNA splicing. The alternatively spliced isoform has an insertion in intron 3, which changes the reading frame and results in a premature termination codon and hences a non-functional protein[58]. Individuals with rs776746 TT genotype are considered to be *CYP3A5* poor metabolizes. Indeed, Subjects carrying this genotype have a poor response to lipid lowering drugs such as statin (atorvastatin, simvastatin, lovastatin) [59], consequently they may develop severe muscle damage linked to this inappropriate treatment[60]. The aforementioned variant is the most frequent and well-studied variant allele of CYP3A5. Its frequency varies widely across human populations [61, 62]. In Tunisian population, it was more similar to the Africans (p.value < 0.05/5*10) and differed significantly to the European and Asian population (p.value > 0.05/5*10). The frequency of rs776746 TT in Tunisian and African were low, it ranged between (0.01 to 0.08). Thus, these populations may have similar statin metabolism and will be of great clinical significance in determining future therapeutic approaches. Regarding the variant rs776746, our findings show that Tunisian population has significant similarities with African populations in predicted atorvastatin response. Yet, they display a distinct profile on PCA. This result could be due to the high heterogeneity of the Tunisian genetic structure composed of European, African, and Asian (Near Eastern) components. For this reason, it seems plausible that for some VIP variant, Tunisians are similar to the Europeans and for others, Tunisians are similar to the Africans. As stated in the study of Mizzi C et al,. 2016, the population groups are distinguished according to the results of PCA or admixture analysis using genotyping data. This approach cannot lead to broad generalizations in the application for VIP variants at the individual level. Although, the approaches aiming to identify clinically relevant actionable VIP variants are conducted in small number of variants. Moreover, this result is in agreement with other studies showing that there are multiple pharmacogenomic profiles across African and non African populations which could affect the safety and efficacy of many therapeutic drugs with CYP3A5 substrate [59, 61]. In addition a considerable heterogeneity in North Africa but not in other geographic regions, has been highlighted revealing a selective pressure on CYP3A5 gene [63]. Based on the rich historic background of Tunisia, we suggest that adaptive T allele of rs776746 might have been introduced by a relatively recent gene flow.

The intronic variant rs12255372, transition 113049143 G>T located on chromosome 10 of *TCF7L2* gene have been thoroughly studied and found to be associated with increased risk of T2DM [64, 65]. The mechanisms by which *TCF7L2* affects susceptibility to the disease remain unraveled. Nevertheless, several studies have shown that decreased *TCF7L2* protein expression inhibits the insulin secretory response to an oral glucose through impaired incretin action[66]. The GODART study performed on Scottish subjects receiving sulfonylurea, showed an association of rs12255372 risk allele with reduced effect of Sulfonylureas hypoglycemic response [65, 66]. The results revealed that the TT patients undergoing early sulfonylurea treatment had approximately two-fold higher probability to fail the sulfonylureas medication (57% versus 17% for TT versus GG respectively)[65]. Conversely, our study shows that the frequency of Tunisians carriers of rs12255372 TT genotype (0.19) is much lower than TSI, ASW, LWK, MKK and CHB (respectively equal to 0.23, 0.22, 0.22, 0.29, 0.29) and much higher than CEU and MEX (0.07, 0.09). Tunisian population seems to be good responder to Sulfonylureas which should be the first line drug for patients, replacing insulin injections. Genotyping the rs12255372 located in *TCF7L2* should be considered when using Sulfonylureas treatment [67].

SNP array designed for the Genome Wide Association Study (GWAS) exploited in our study do not allow to fully assess the contributions of variants implicated in MetS drugs response due to the non-uniform coverage of all the chromosomal regions. Indeed, specialised pharmacovariants chip like DMET array increases the power to identify common and rare variations validated for their involvement in drugs metabolism [68–70].

The current clinical pharmacogenomics practice considered that inter-individual drug response variability is mainly based on genetic common variants [1, 3, 46, 47, 71]. For this reason, in our study, we have focused on common variants among Tunisian compared to other populations. Nevertheless, it is well known that rare variants are expected to have more effects on response to drug than common variants because they will not have been subject to purifying selection after the recent expansion of the human population. [72, 73].

## Conclusion

The present study showed that Tunisian population is genetically heterogeneous regarding the studied pharmacogenes involved in the response to the MetS components. The allelic and genotypic frequencies do not differ homogenously among the components. This shows the complexity of the genetic components of response to treatment in admixed populations. This study should be extended to other North African population to take into account their peculiarities in order to effectively orient dose and drug prescription to ovoid serious adverse reactions.

## Supporting information

S1 Fig Principal component analysis of the Tunisian subpopulation and worldwide populations. The plot reveals three distinct clusters showing that the Tunisian population present a close affinity with the North Africans and Europeans and distinct from South Africans and Asians. Tunisian population; Capital Tunis TU_TC, coastal city of Monastir TU_MC (AffymetrixChip 6.0 genotyping array), African ancestry in the south Western USA (ASW); a northwestern European population (CEU); the Han Chinese in Beijing, China (CHB); a Chinese population of metropolitan Denver, Colorado, USA (CHD); the Gujarati Indians in Houston, Texas, USA (GIH); the Japanese population in Tokyo, Japan (JPT); the Luhya people in Webuye, Kenya (LWK); people of Mexican ancestry living in Los Angeles, California, USA (MEX); the Maasai people in Kinyawa, Kenya (MKK); the Tuscan people of Italy (TSI); and the Yoruba in Ibadan, Nigeria (YRI); data from HapMap: ftp://ftp.ncbi.nlm.nih.gov/hapmap/ and Algeria (ALG), Egyptia (EGY), Libya (LIB), Tunisia Dwiret TUN_Ber, Lebanon (LIB), Morocco South (MCS), Morocco North (MCN), Spain South (SPS), Spain North (SPN), Spain Basc (SBA),: Sub-Saharan (SAH), Canary Island (CIS); data from the literature.
(TIFF)

S2 Fig Distruct barplot result. The figure shows the different bar plots according to the different K number.
(DOCX)

S3 Fig Best_K_By_Evanno-Delta K By K graph. The graph shows the best K equal to 3 according to delta K as proposed by Evanno.
(TIF)

S4 Fig Analysis pipeline. (TIF)

S1 Table Genotype frequencies of clinical relevant VIP variant in Tunisia an ten HapMap populations. The table reveals the similarities or divergences of the Tunisian population to the other studied populations. This table highlight that the genotype frequencies of VIP variants significantly affect a population’s response to a given drug.
(XLSX)
