# Supplementary figures and images for: Pharmacogenetic landscape of Metabolic Syndrome components drug response in Tunisia and comparison with worldwide populations
Source: PLoS One. 2018 Apr 13;13(4):e0194842. doi: 10.1371/journal.pone.0194842 (PMC5898725; doi:10.1371/journal.pone.0194842)

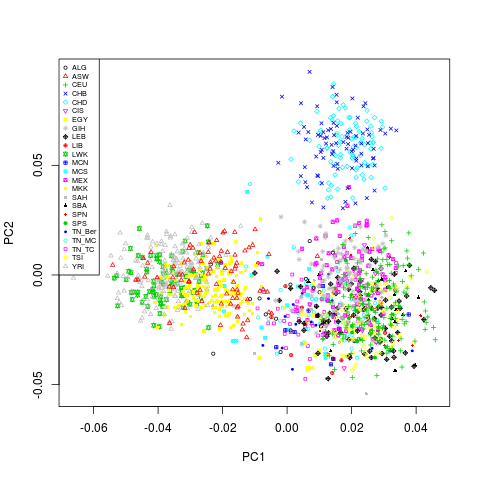

Supplement: S1 Fig — The plot reveals three distinct clusters showing that the Tunisian population present a close affinity with the North Africans and Europeans and distinct from South Africans and Asians. Tunisian population; Capital Tunis TU_TC, coastal city of Monastir TU_MC (AffymetrixChip 6.0 genotyping array), African ancestry in the south Western USA (ASW); a northwestern European population (CEU); the Han Chinese in Beijing, China (CHB); a Chinese population of metropolitan Denver, Colorado, USA (CHD); the Gujarati Indians in Houston, Texas, USA (GIH); the Japanese population in Tokyo, Japan (JPT); the Luhya people in Webuye, Kenya (LWK); people of Mexican ancestry living in Los Angeles, California, USA (MEX); the Maasai people in Kinyawa, Kenya (MKK); the Tuscan people of Italy (TSI); and the Yoruba in Ibadan, Nigeria (YRI); data from HapMap: ftp://ftp.ncbi.nlm.nih.gov/hapmap/ and Algeria (ALG), Egyptia (EGY), Libya (LIB), Tunisia Dwiret TUN_Ber, Lebanon (LIB), Morocco South (MCS), Morocco North (MCN), Spain South (SPS), Spain North (SPN), Spain Basc (SBA),: Sub-Saharan (SAH), Canary Island (CIS); data from the literature. (TIFF) [file pone.0194842.s001.tiff]

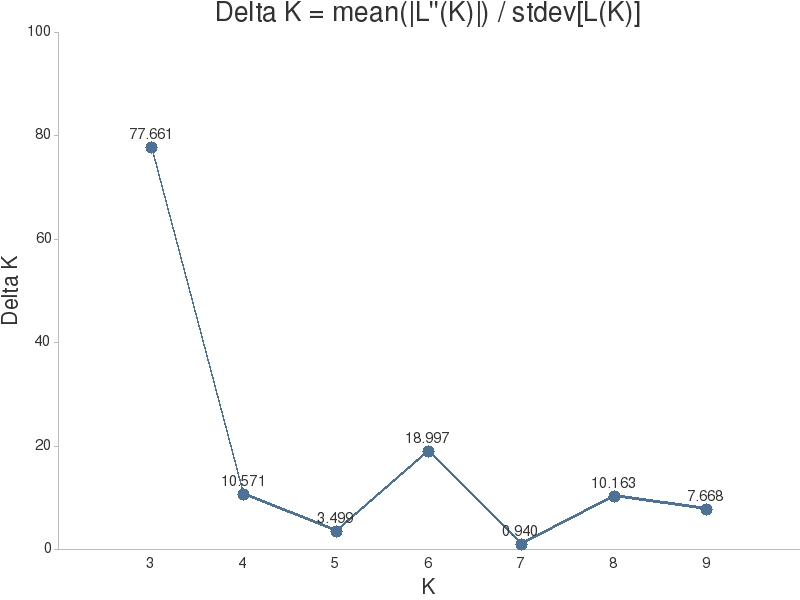

Supplement: S3 Fig — The graph shows the best K equal to 3 according to delta K as proposed by Evanno. (TIF) [file pone.0194842.s003.tif]

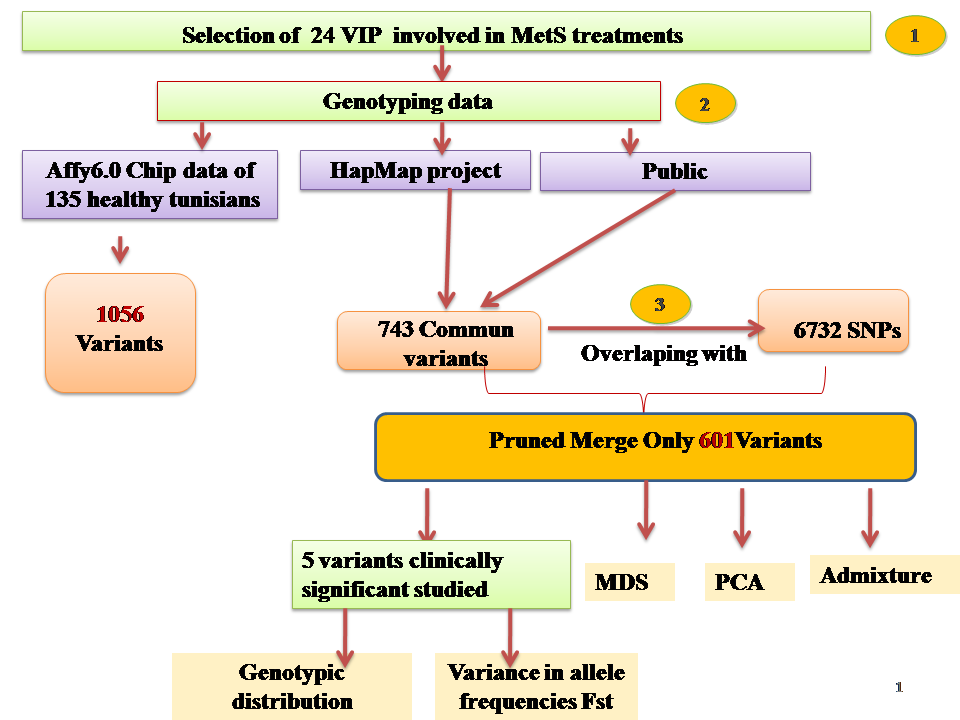

Supplement: S4 Fig — (TIF) [file pone.0194842.s004.TIF]
