# Supplementary material for: Pharmacogenetic landscape of Metabolic Syndrome components drug response in Tunisia and comparison with worldwide populations
Source: PLoS One. 2018 Apr 13;13(4):e0194842. doi: 10.1371/journal.pone.0194842 (PMC5898725; doi:10.1371/journal.pone.0194842)

CLUMPAK Distruct for many K's - Job 1485296552 summary

Distruct output images:

K=2 sucfin-run-1-f


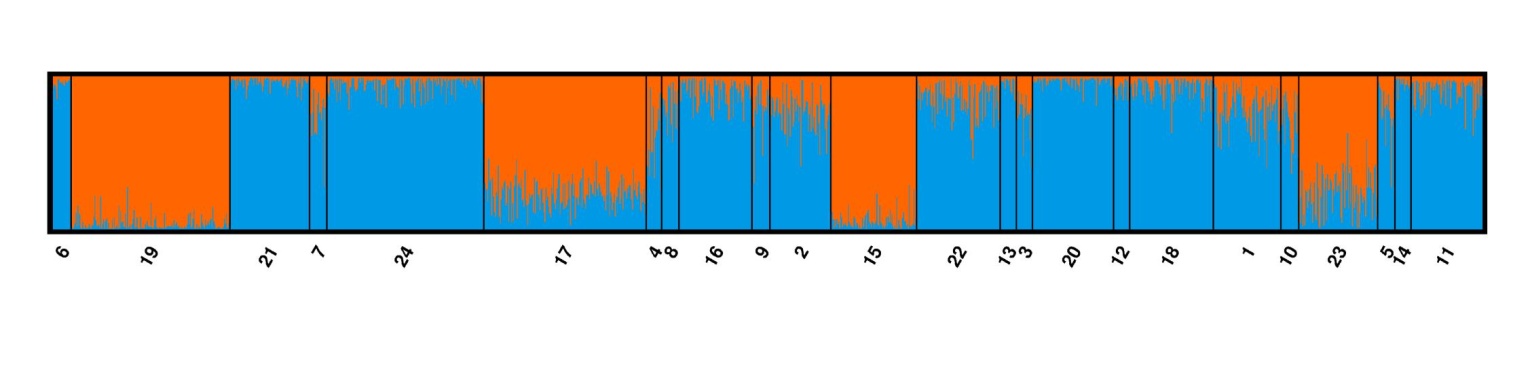


K=2 sucfin-run-2-f


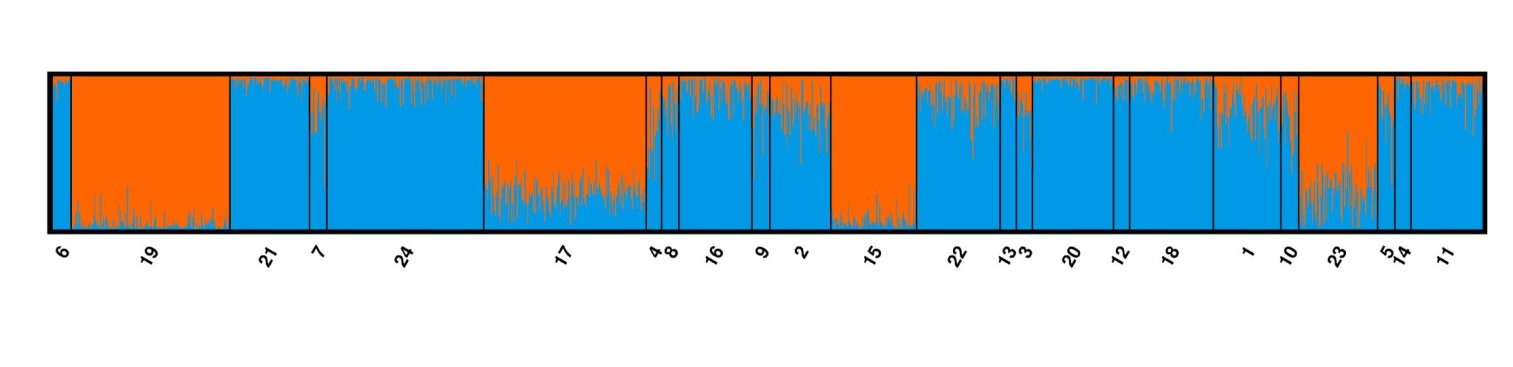


K=2 sucfin-run-3-f


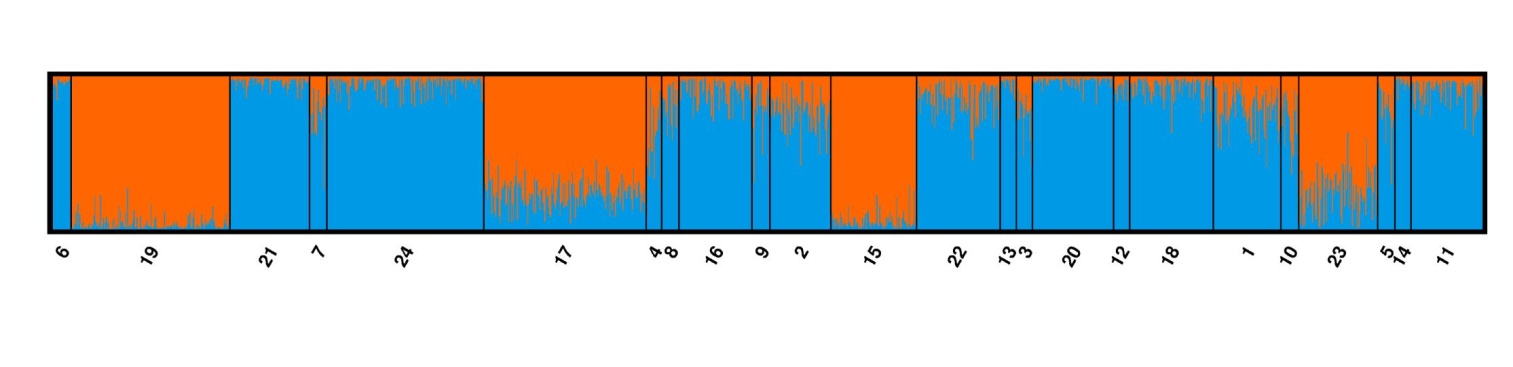


K=3 sucfin-run-4-f


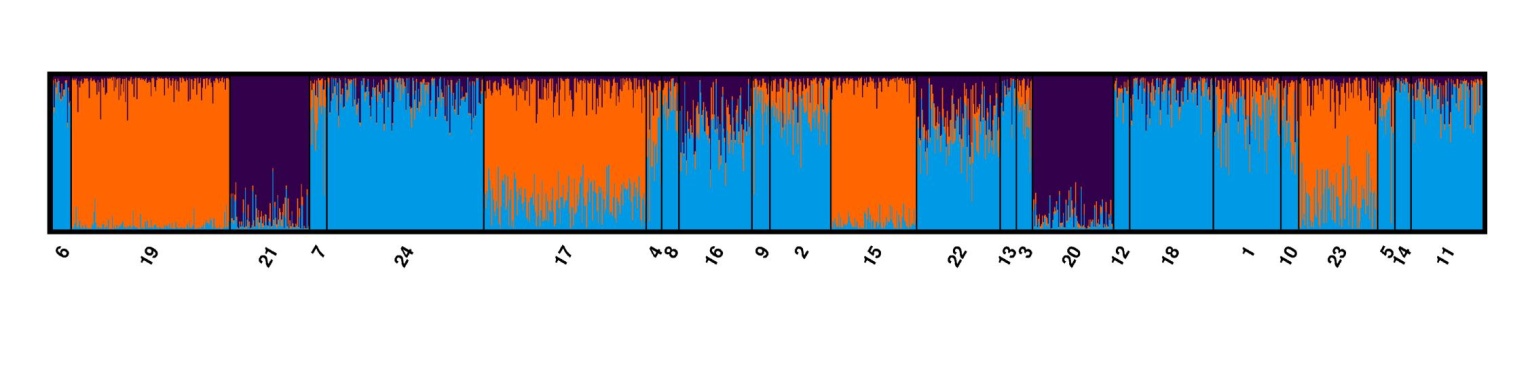


K=3 sucfin-run-5-f


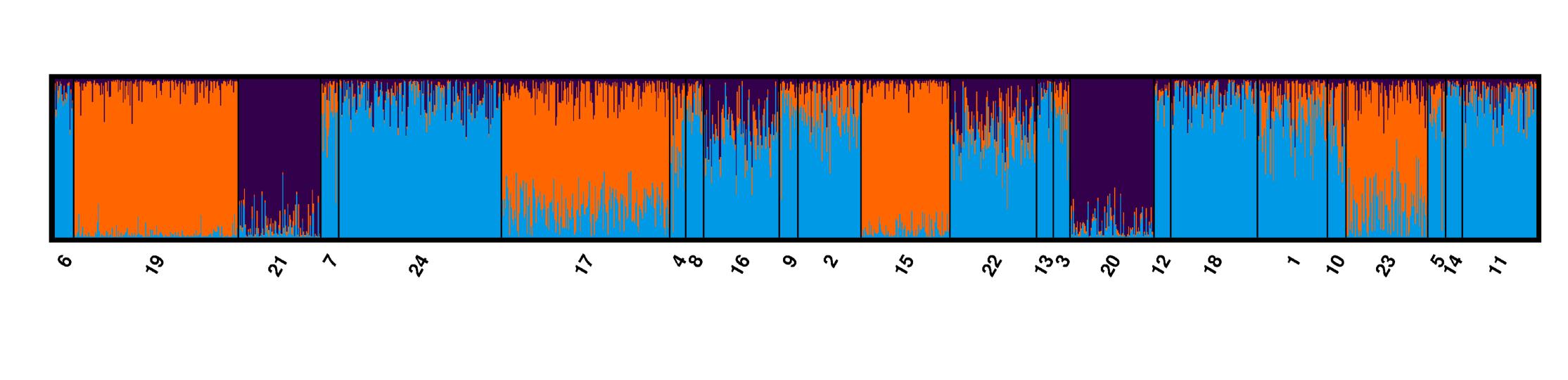


K=3 sucfin-run-6-f


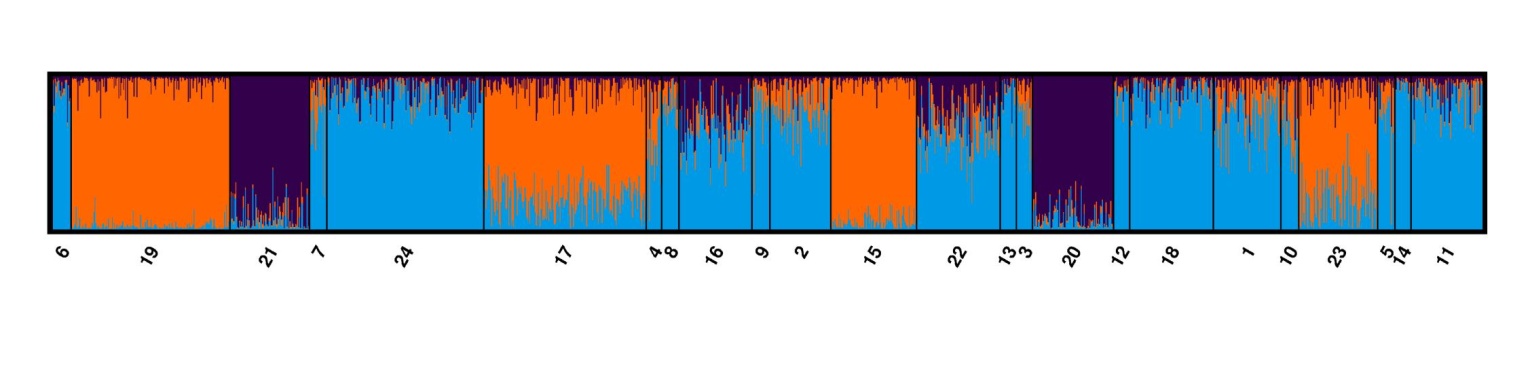


K=4 sucfin-run-7-f


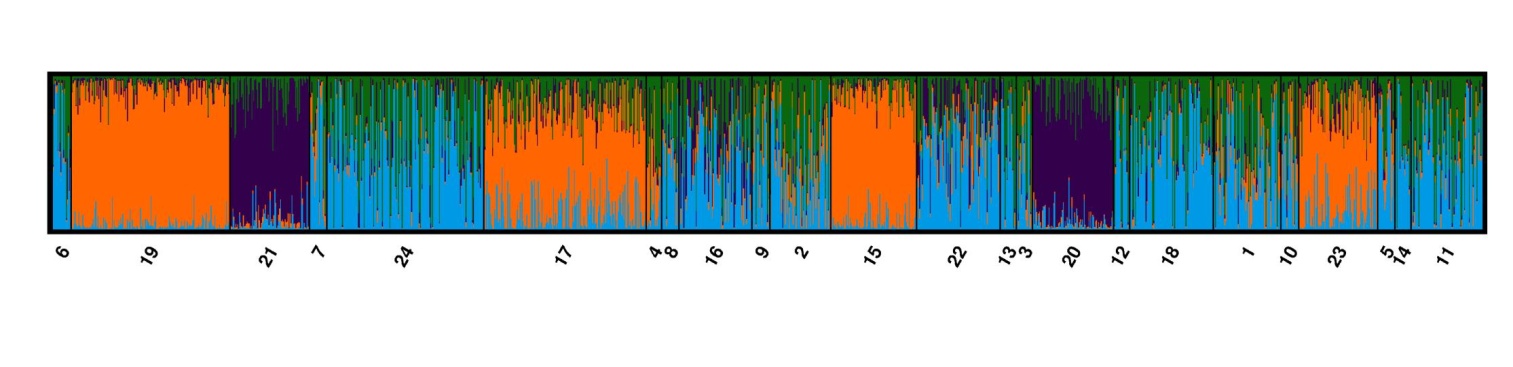


K=4 sucfin-run-8-f


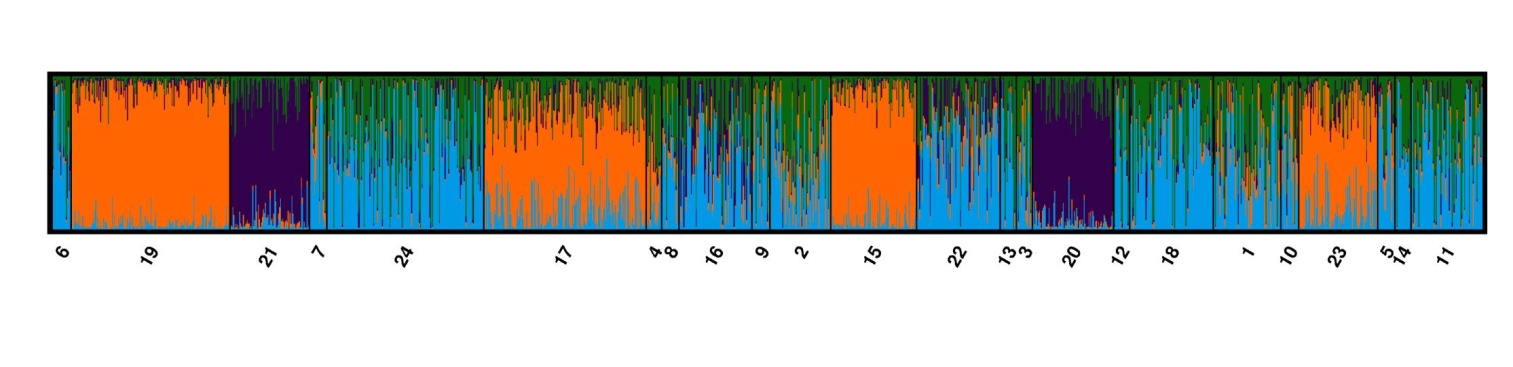


K=4 sucfin-run-9-f


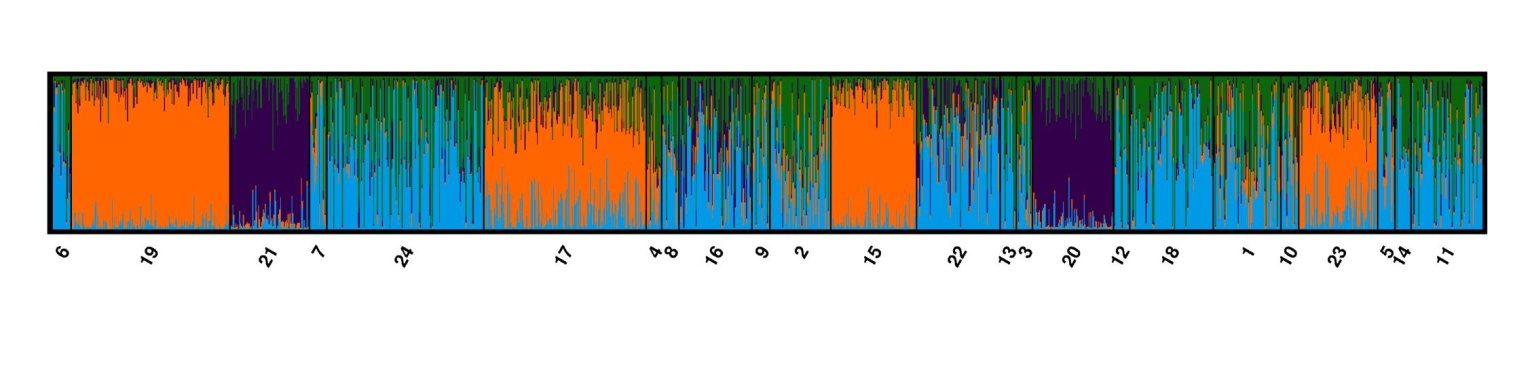


K=5 sucfin-run-10-f


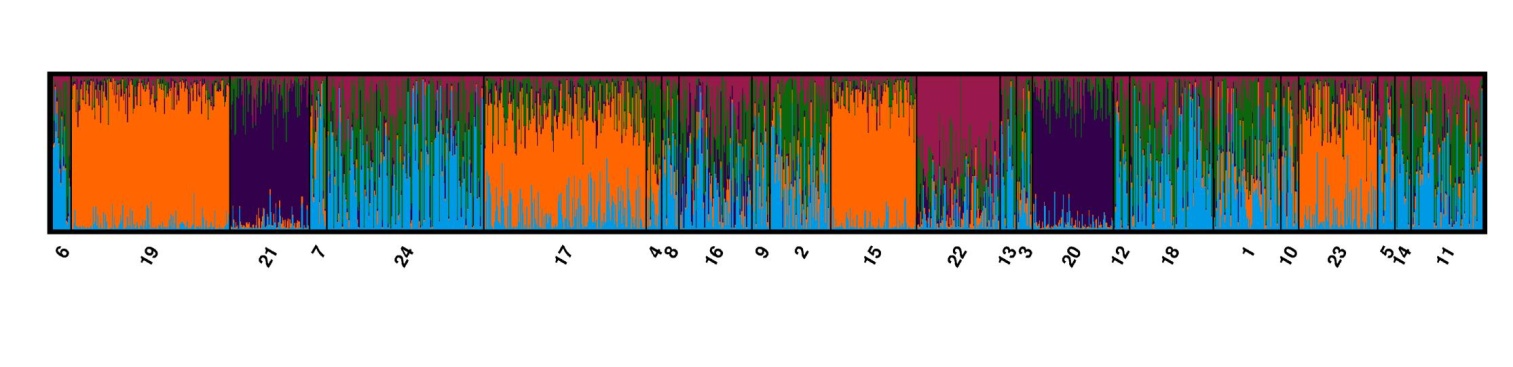


K=5 sucfin-run-11-f


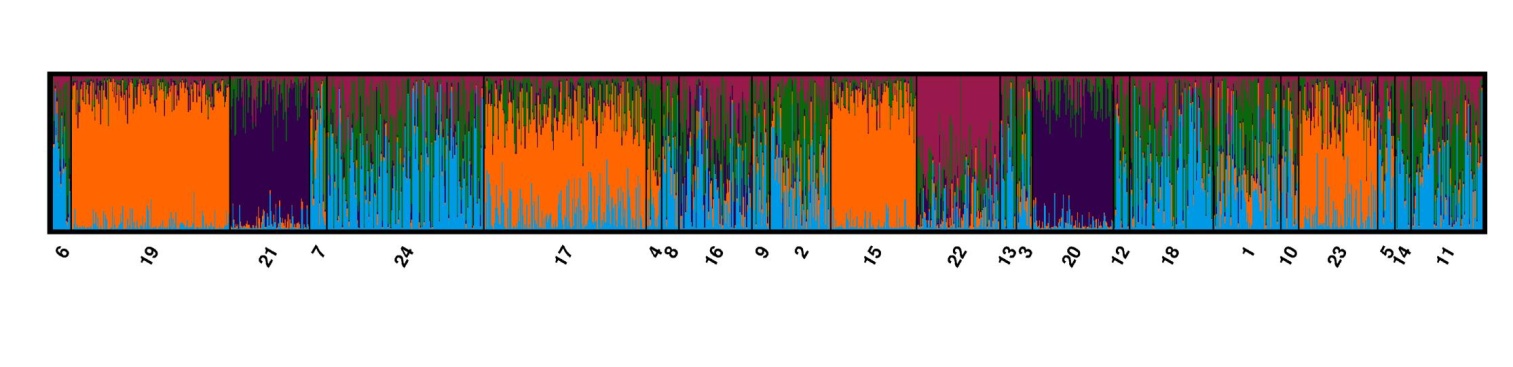


K=5 sucfin-run-12-f


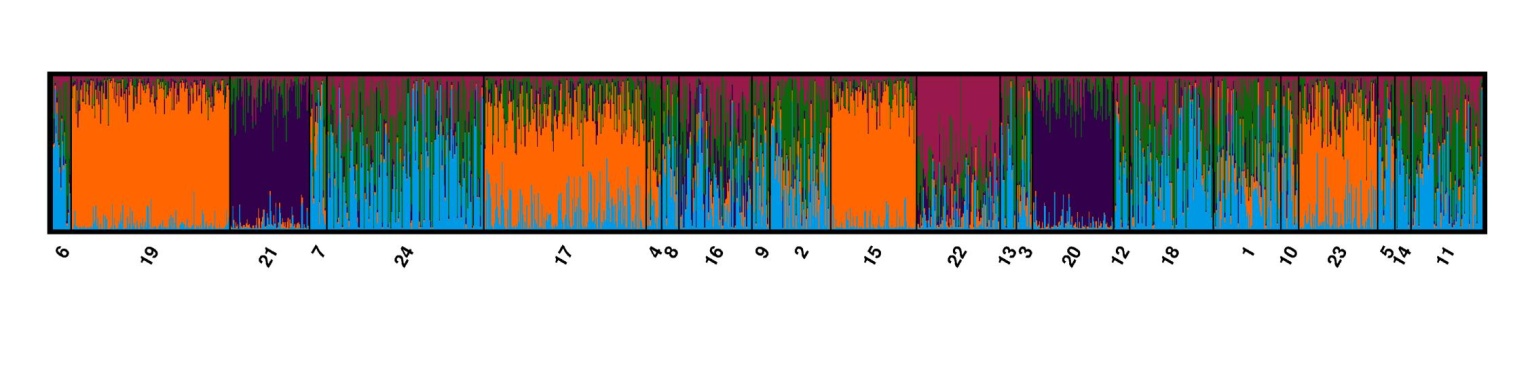


K=6 sucfin-run-13-f


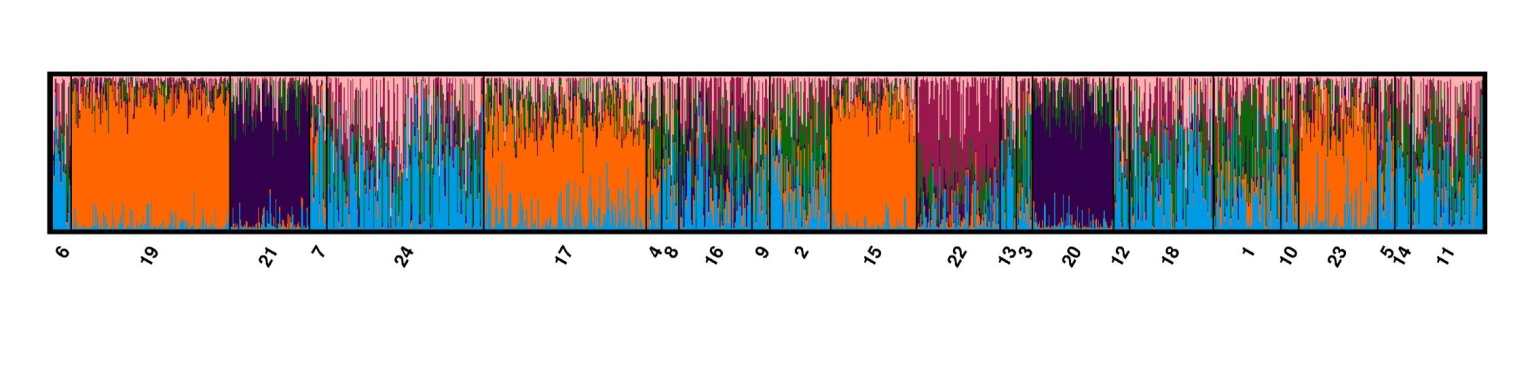


K=6 sucfin-run-14-f


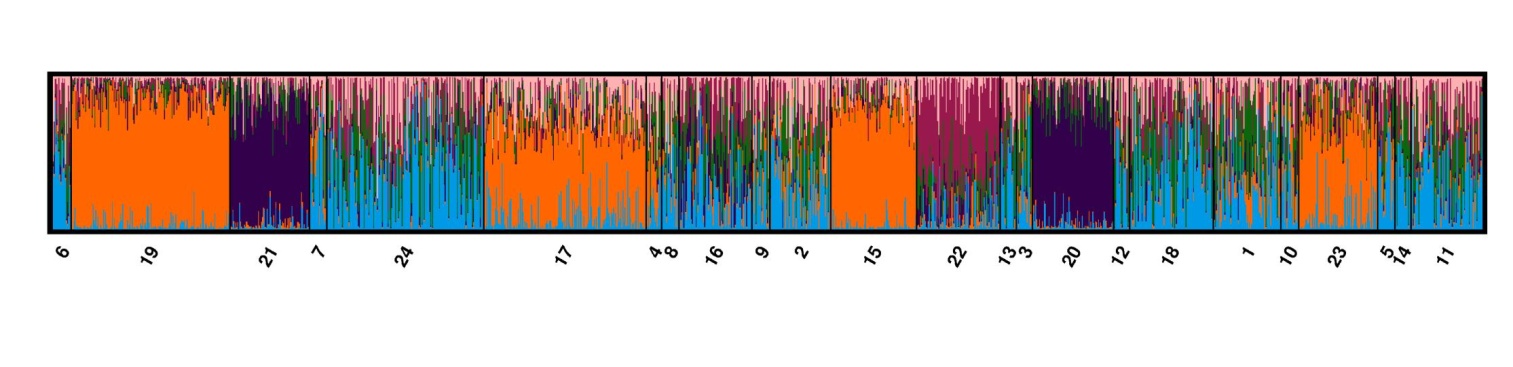


K=6 sucfin-run-15-f


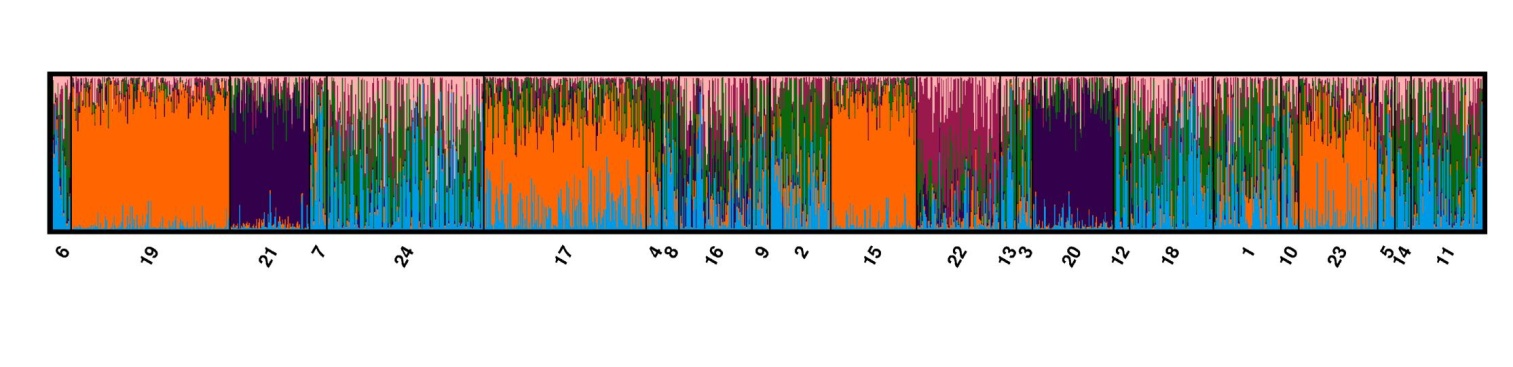


K=7 sucfin-run-16-f


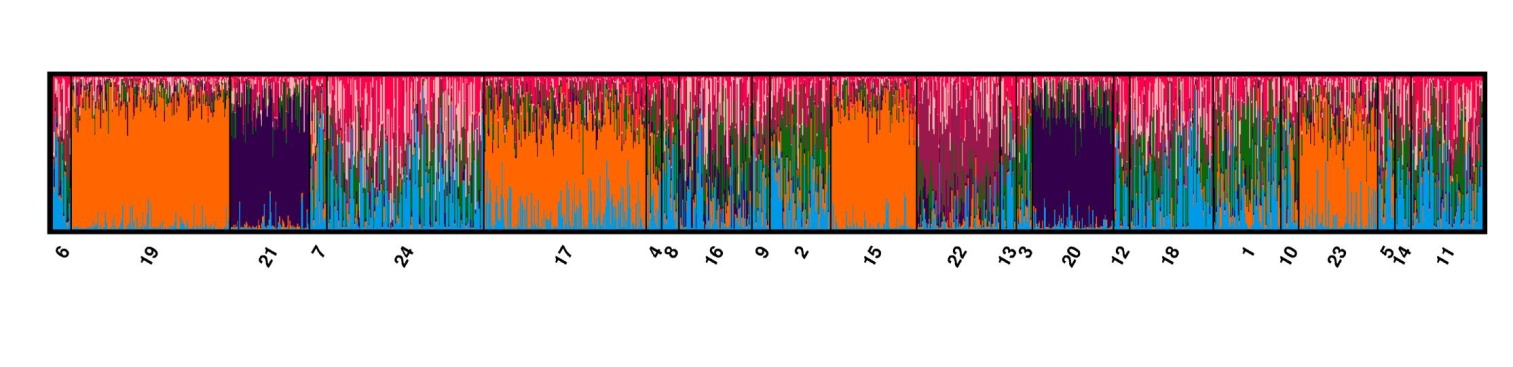


K=7 sucfin-run-17-f


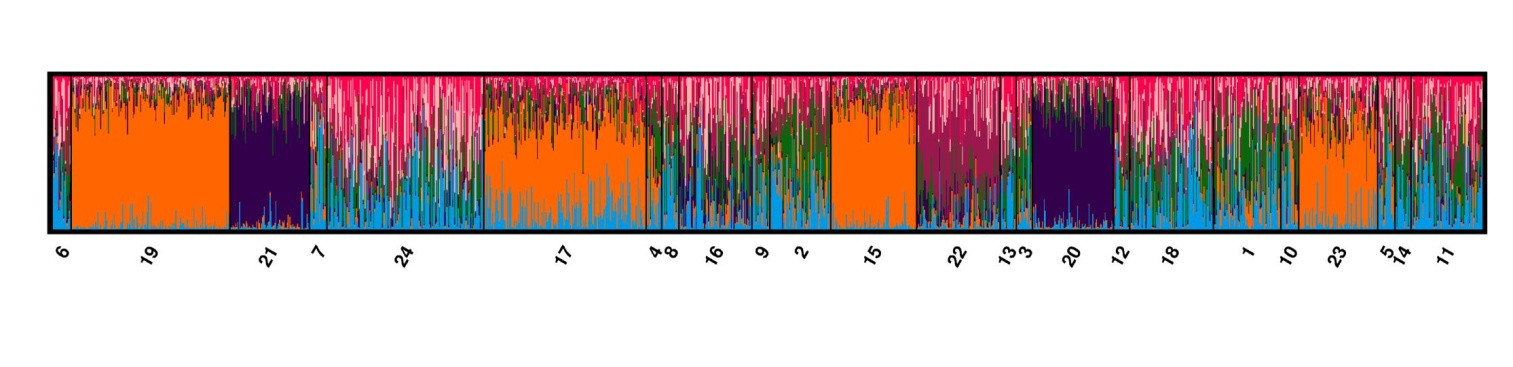


K=7 sucfin-run-18-f


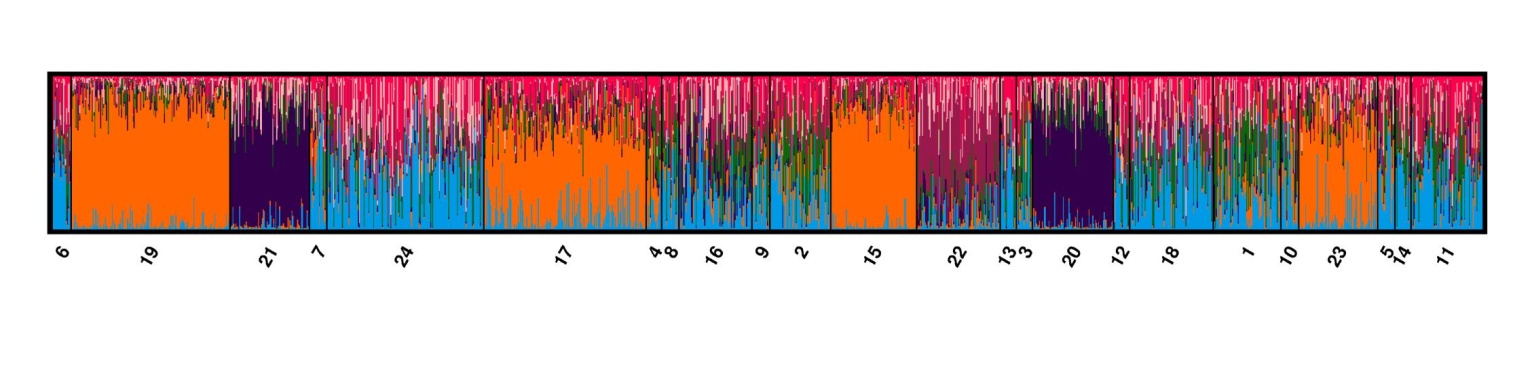


K=8 sucfin-run-19-f


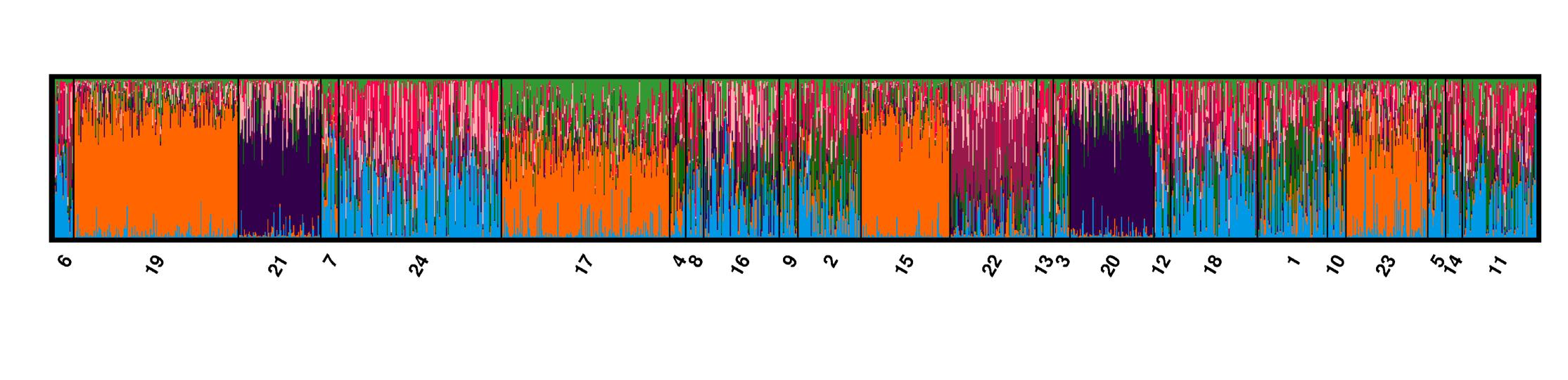


K=8 sucfin-run-20-f


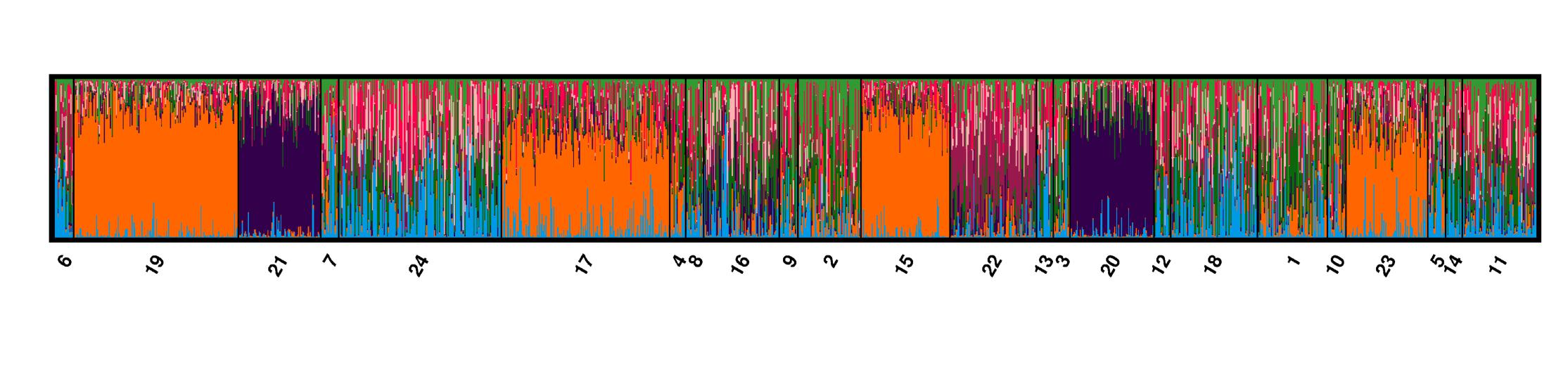


K=8 sucfin-run-21-f


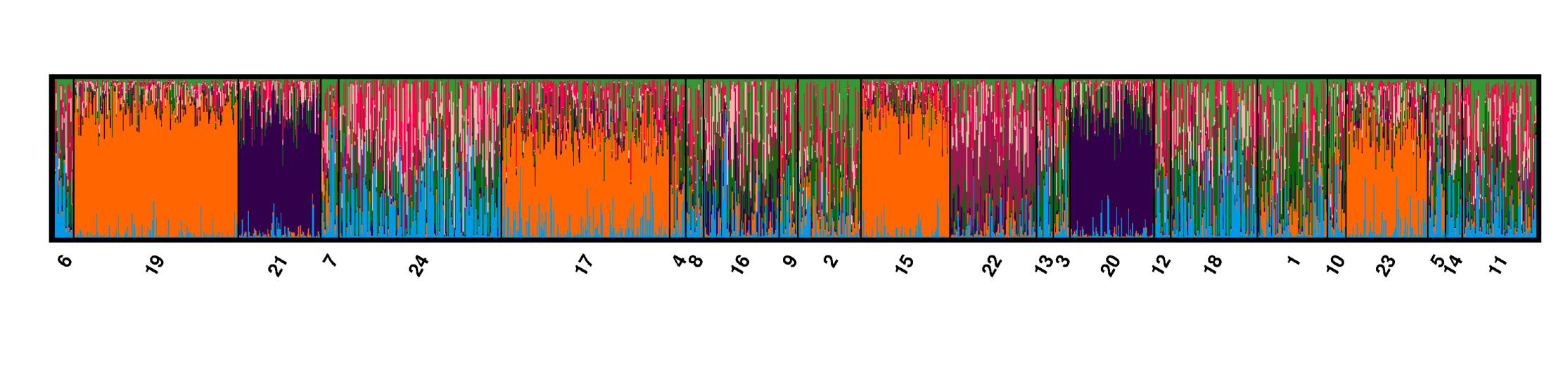


K=9 sucfin-run-22-f


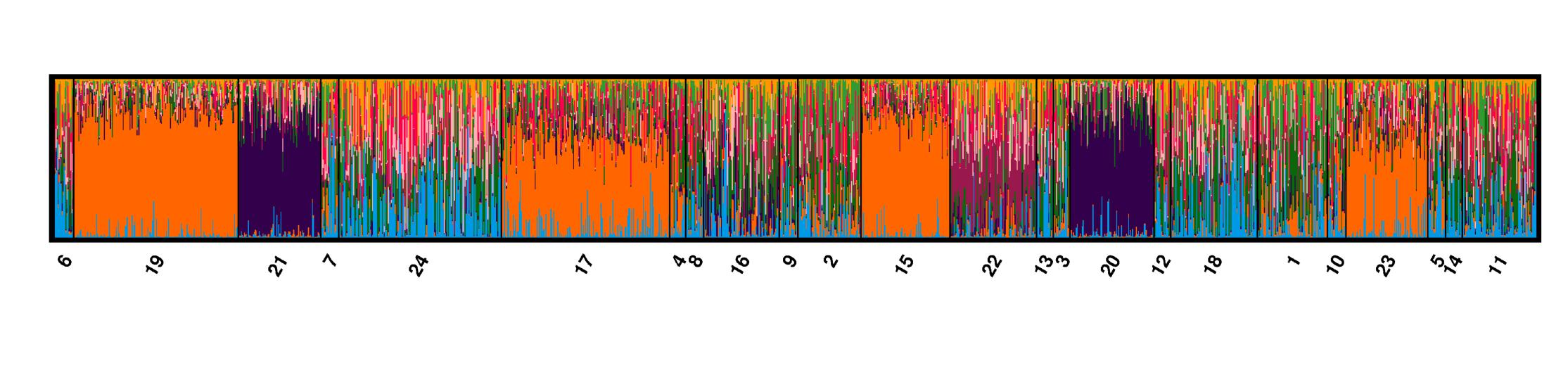


K=9 sucfin-run-23-f


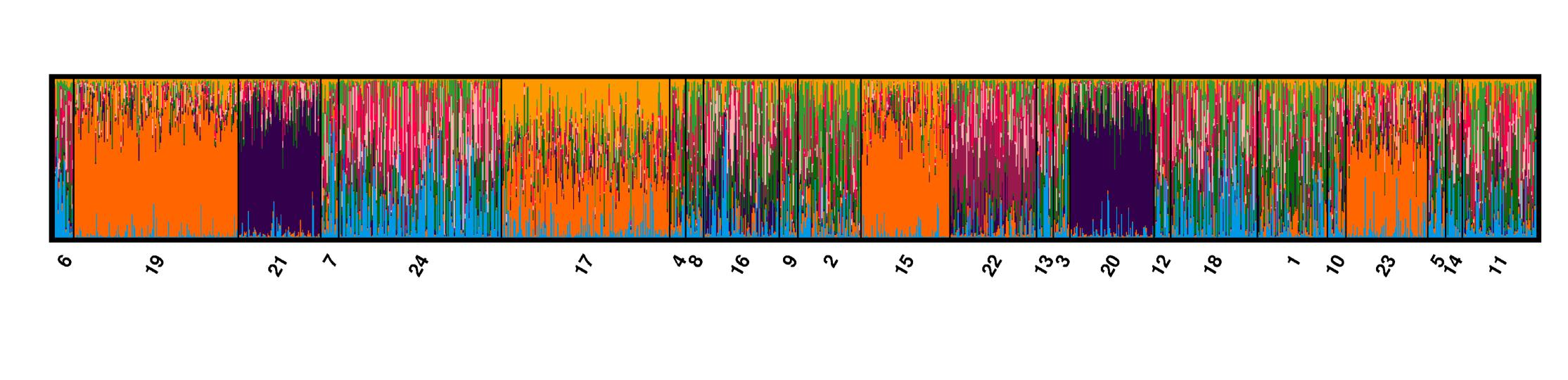


K=9 sucfin-run-24-f


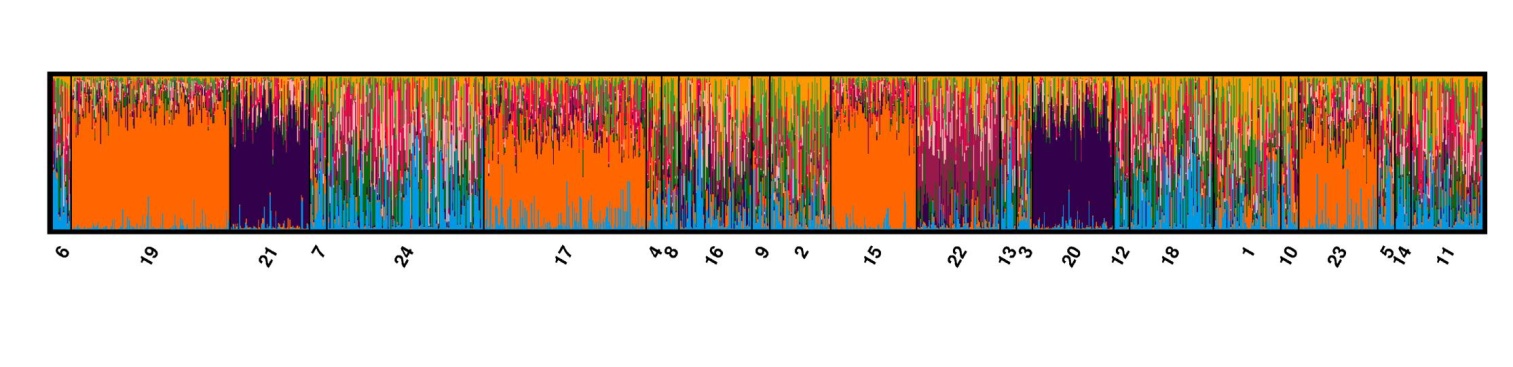


K=10 sucfin-run-25-f


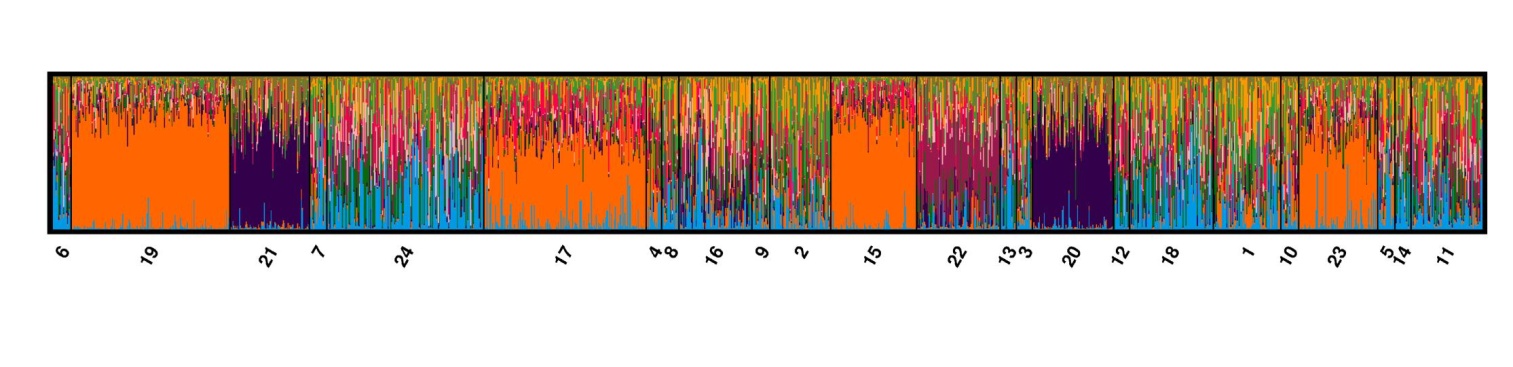


K=10 sucfin-run-26-f


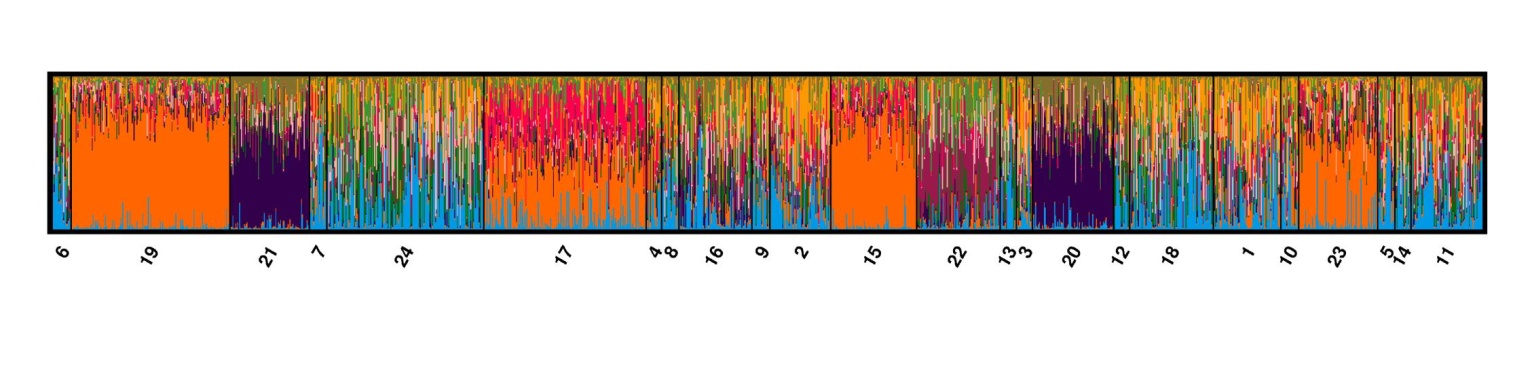


K=10 sucfin-run-27-f


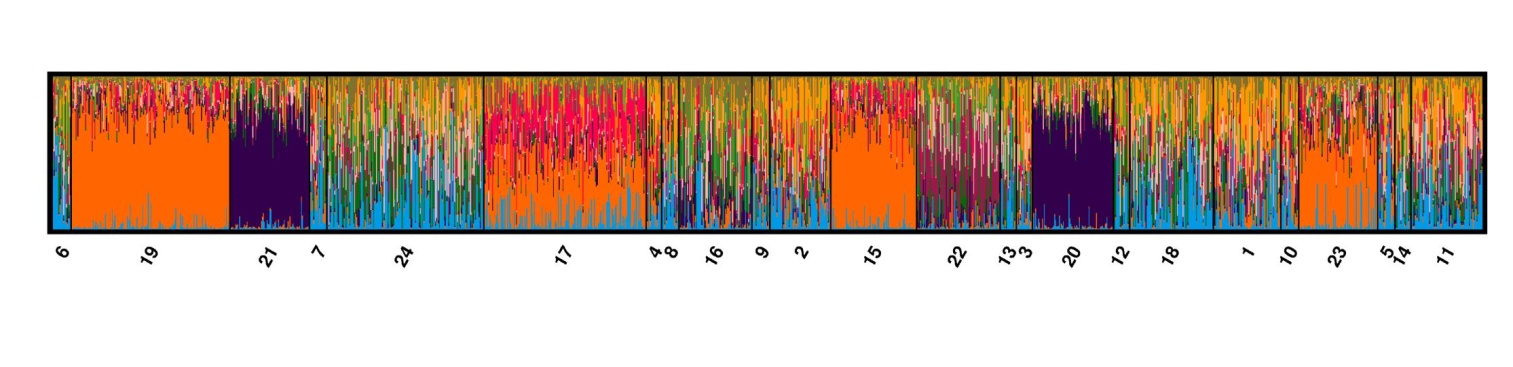

Supplement: S2 Fig — The figure shows the different bar plots according to the different K number. (DOCX) [file pone.0194842.s002.docx]
